# The snakehead retrovirus promoter functions independently of the 3’ORF protein and its products are maternally inherited in transgenic zebrafish

**DOI:** 10.1371/journal.ppat.1013243

**Published:** 2025-06-12

**Authors:** Rachel Zamostiano, Odelia Pisanty, Japhette Esther Kembou-Ringert, Reem Abu Rass, Avi Eldar, Marcelo Ehrlich, Yoav Gothilf, Eran Bacharach

**Affiliations:** 1 The Shmunis School of Biomedicine and Cancer Research, The George S. Wise Faculty of Life Sciences, Tel Aviv University, Tel-Aviv, Israel; 2 School of Neurobiology, Biochemistry and Biophysics, The George S. Wise Faculty of Life Sciences, Tel-Aviv University, Tel-Aviv, Israel; 3 Department of Virology, Kimron Veterinary Institute, Bet Dagan, Israel; 4 Sagol School of Neuroscience, Tel-Aviv University, Tel-Aviv, Israel; University of Wisconsin, UNITED STATES OF AMERICA

## Abstract

The exogenous snakehead retrovirus (SnRV) is an unclassified member of the Orthoretrovirinae subfamily, discovered in cell lines derived from several fish species. SnRV resembles complex lentiviruses and potentially encodes accessory proteins, including the product of the 3’ open reading frame (3’ORF). The 3’ORF protein was suggested to function as a transactivator of transcription (Tat). Here, we constructed an infectious molecular clone for SnRV and tested the effects of 3’ORF mutations on SnRV transcription. Although replacing 3’ORF with foreign sequences strongly reduced virus expression and production, an out-of-frame point mutation in 3’ORF had only a minimal effect on SnRV replication. This latter result suggests that the 3’ORF protein does not function as Tat and that SnRV transcription is largely independent of the product of this ORF. We also show that *in vitro*, the SnRV promoter is versatile and robustly functioning in both fish and mammalian cultured cells. Finally, the SnRV promoter was transiently active in injected zebrafish embryos as early as the blastula stage. In transgenic zebrafish, this promoter drives enhanced expression in sensory organs and gonads, and its generated products are maternally inherited. Considering these characteristics, the SnRV promoter emerges as a promising candidate for developing versatile expression vectors applicable to research and biotechnological applications.

## Author summary

Retroviruses are genetic elements that can insert themselves into the DNA of host cells, and they play important roles in biology and biotechnology. The fish snakehead retrovirus (SnRV) was suggested to enhance its own gene transcription via a protein encoded by its 3’ open reading frame (3’ORF) in a similar way to the Tat protein in other complex retroviruses (e.g., the human immunodeficiency virus - HIV). To test this hypothesis, we created lab clones of SnRV and studied how disrupting the 3’ORF affects the gene expression and replication of the virus. We found that the 3’ORF protein is not essential for SnRV replication, showing that viral transcription occurs independently of this protein. We also discovered that the SnRV’s promoter functions robustly in both fish and mammalian cells. In injected zebrafish embryos, it is active as early as the blastula stage, and in transgenic zebrafish, it drives strong gene expression in sensory organs and gonads. Moreover, the promoter’s expression products in oocytes are maternally inherited to the offspring. These findings have implications for the field of retroviral evolution, and highlight the SnRV promoter’s potential as a versatile tool for scientific research and biotechnology.

## Introduction

The snakehead retrovirus (SnRV), a member of the Orthoretrovirinae subfamily [1], was discovered in cell lines derived from southeast Asian freshwater fish by Onions’ group [2]. Specifically, electron microscopy revealed morphologically similar C-type virus particles in cell lines derived from two striped snakehead fish (*Ophicephalus striatus*, each captured at different times and locations), one climbing perch (*Anabas testudineus*), and one snakeskin gourami (*Trichogaster pectoralis*). Particles from all four sources possessed Mn^2+^-dependent reverse transcriptase (RT) activity, were similarly separated in sucrose density gradients, and induced cytopathic effects in cultures of the BF-2 cell line derived from bluegill fry (*Lepomis machrochirus*).

The SnRV genome, expressed in one of the above cell lines (the SSN-1 cell line, derived from the striped snakehead fish), was sequenced and annotated (GenBank U26458.1, and see schematic map below) [3]. This analysis revealed features resembling complex retroviruses: (*i*) a relatively long (136 residues) cytoplasmic tail of the predicted envelope protein - a size reminiscent of the long envelope tails of lentiviruses [4]. (*ii*) The 11.2-kb SnRV provirus has complex splicing patterns resembling the ones of spumaviruses and two lentiviruses - the bovine leukemia virus and the human T-cell leukemia virus. (*iii*) Like these viruses, SnRV potentially encodes accessory/regulatory proteins in addition to the Gag, Pol, and Env precursors. Specifically, sub-genomic mRNAs harbor four open-reading frames - ORF1, ORF2, and ORF3 for proteins with sizes of 52, 94, and 76 residues, respectively, and a larger ORF (205 codons), named 3’ ORF (henceforth, ‘3’ORF’), which its first 14 residues overlap the *env* gene end with a different reading frame. Accessory proteins are also encoded by the complex fish retroviruses Walleye dermal sarcoma virus (WDSV) and the two types of the Walleye epidermal hyperplasia virus (WEHV-1 and WEHV-2), which cause dermal sarcoma and discrete epidermal hyperplasia, respectively (reviewed in [5]). These three viruses comprise the *Epsilonretrovirus* genus within the *Orthoretrovirinae* subfamily, while the SnRV is an unclassified subfamily member [1]. Currently, no known disease is attributed to SnRV [5]. Notably, SnRV is the only exogenous representative of the SnRV-clade of the *Orthoretrovirinae* - a clade composed of SnRV and endogenous retroviruses (ERVs) detected in reptiles, birds, amphibians, and fish lineages [6–8].

It has been suggested that despite the lack of significant sequence similarities, the SnRV 3’ORF product functions as a transcriptional transactivator, similar to the Tat proteins of lentiviruses [3]. This hypothesis was based on features shared by the 3’ORF product and the Tat proteins: an N-terminal acidic region and a cysteine cluster followed by a basic region [9]. The Tat protein is an essential factor for the transcription of the human immunodeficiency virus type 1 (HIV-1), enhancing both transcriptional initiation and elongation (reviewed in [10]). HIV-1 Tat binds an RNA stem-loop (the trans-activation response element; TAR) located at the 5’ ends of nascent viral transcripts and recruits the cellular CDK9 and cyclin T1 proteins (as part of the positive transcription elongation complex; P-TEFb) to phosphorylate the RNA polymerase II and two associated elongation regulatory factors (NELF and DSIF). This phosphorylation relieves the promoter-proximal stalling of the RNA polymerase II and increases transcriptional processivity. In addition, Tat recruits to the HIV-1 promoter (the 5’ Long Terminal Repeat; LTR), the P-TEFb, and parts of the pre-initiation complex of transcription, including the mediator complex components and the TATA-binding protein (TBP). Thus, the presence of Tat enhances transcriptional initiation from the LTR [11–13] and the elongation of the nascent transcripts [14–16]. In line with its crucial role in transcriptional regulation, Tat-deletion HIV-1 mutants failed to activate their transcription and to express the Gag and envelope proteins [16,17].

Here, we constructed an infectious molecular clone for SnRV and tested the effects of 3’ORF mutations on SnRV transcription. Replacing the 3’ORF sequence with foreign sequences of comparable sizes dramatically reduced virus expression and production. However, frameshifting 1 bp insertion in the 3’ORF had only a minimal effect on SnRV expression and replication. These results suggest that the 3’ORF sequence may act in *cis*, but the product of this ORF is not functioning as a Tat protein, and, accordingly, the SnRV promoter activity is largely independent of this product. We also show that the SnRV promoter is versatile, functioning in both fish and mammalian cultured cells. Finally, we demonstrate that in transgenic zebrafish, this promoter is robustly active in sensory organs and gonads, and that SnRV promoter-generated products are maternally inherited.

## Results

### Construction of a SnRV molecular clone

**Fig 1:**
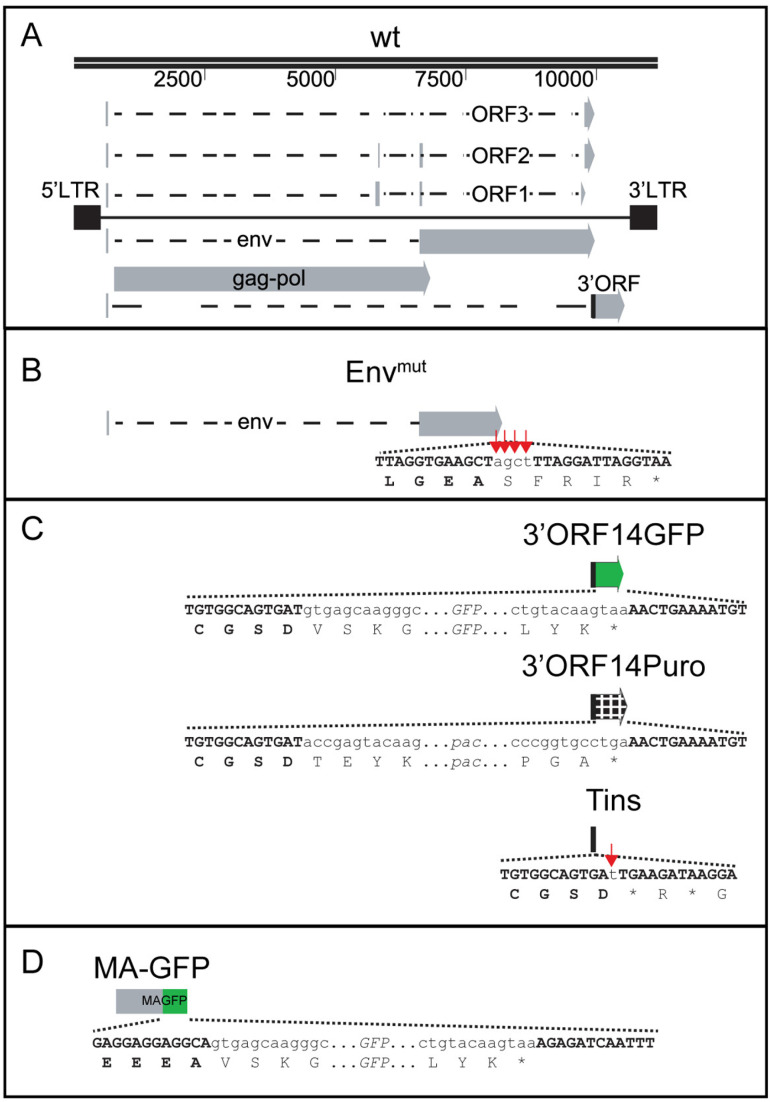
Schematics of the wt and mutants SnRV clones. (A) Scaled schematics of SnRV wt clone, based on [3]. Black boxes, 5’ and 3’ LTRs. Dashed lines, introns. Gray arrows and bars, exons. Black bar, the first 14 residues of 3’ORF, overlapping the env ORF. For simplicity, only one of the two putative 3’ORF mRNAs (transcript *c*; [3]) is shown. Upper line, map scale (in nucleotides). (B-D) SnRV mutants. The mutations’ location is portrayed relative to the map in (A). The schemes depict the junctions between the SnRV nucleotides and cognate amino acids (uppercase and bold letters) and the nested non-viral sequences (nucleotides in non-bold lowercase letters). Red arrows mark four nucleotides in the Env^mut^ clone (B) and a single nucleotide in the Tins clone (C), inserted to make out-of-frame mutations. Asterisks, stop codons. Green boxes (C, D) and a grid (C) illustrate the GFP and the pac ORFs, respectively.

To clone SnRV provirus, we PCR amplified overlapping sequences that spanned the entire SnRV genome, using the genomic DNA of E-11 cells as a template, as these cells are chronically infected with SnRV [2,18–21]. The PCR products harbor overlapping termini, allowing their co-assembly and cloning into the pGREG525 plasmid [22] through homologous recombination in yeast. Next, we generated a consensus sequence by comparing the sequences of the cloned SnRV genome, the published SnRV sequence (GenBank U26458.1) [3], and a SnRV sequence that we have obtained by high-throughput sequencing (HTS) of SnRV virion RNA (Materials and Methods). We then modified the cloned nucleotides to match the consensus sequence. Overall, the cloned/consensus sequence deviated from the published 10,688 nucleotides of the GenBank sequence by 21 nucleotides. These 21 changes included three located in the noncoding region downstream of the primer binding site and upstream of the Gag ORF and 18 nonsynonymous substitutions scattered over the Gag, Pol, Env, and ORF2 sequences (detailed in S1 Table). Notably, the LTR and 3’ORF sequences of the three sequencing sources fully matched. We named the SnRV clone ‘SnRV wt’ (Fig 1A depicts its genome organization).

An independent authentication of several nonsynonymous substitutions, present in the SnRV wt clone but not in the GenBank U26458.1 sequence, came from mass spectrometry analyses of the parental SnRV virions purified from E-11 cells. Specifically, we re-examined the results of former mass spectrometry analyses that we had performed on pellets of tilapia lake virus (TiLV) virions, purified from E-11 culture supernatants [23], as TiLV and SnRV virions co-purify by ultracentrifugation through sucrose cushions [20]. Indeed, we identified 329 peptides derived from SnRV Gag, Pol, or Env proteins in these records. Of these, nine peptides overlapped the locations of six nonsynonymous substitutions, and they all fully matched the sequence of the SnRV wt clone (S1 Table).

### SnRV wt clone is infectious and induces cytopathic effects in BF-2 cells

**Fig 2:**
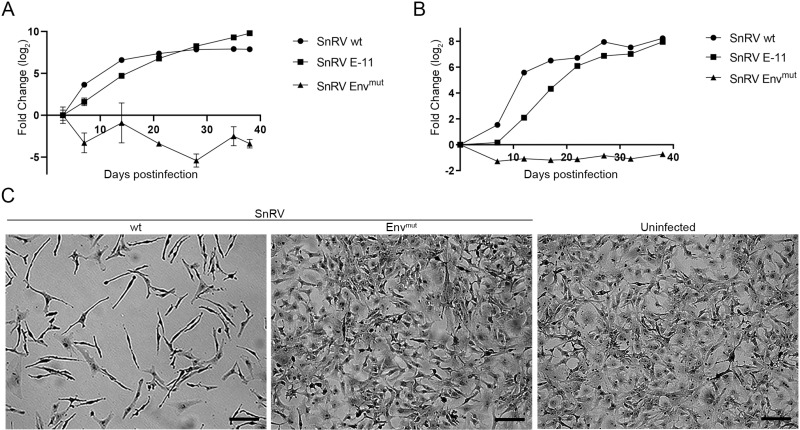
SnRV wt molecular clone is infectious. (A, B) Kinetics of SnRV spread. BF-2 cells were infected with equal amounts - normalized by qRT-PCR with SnRV *pol*-derived primers (A) or RT activity (B) - of SnRV wt, SnRV Env^mut^, or SnRV from E-11 cultures (SnRV E-11). On the indicated days postinfection, the culture supernatants were sampled (140 µl) to quantify viral RNA levels by qRT-PCR (A), or harvested (10 ml) for pelleting the virions by ultracentrifugation to determine the pellets’ RT activity by SG-PERT (B). The fold change (log_2_) between each time point and (A) the initial time point (day 3 postinfection) or (B) the background sample (a pellet of a supernatant of naïve BF-2 culture) was calculated. Values represent the mean ± SD of three technical repeats. (C) Phenotypic changes in SnRV-infected BF-2 cells. Sub-confluent BF-2 cultures were infected (SnRV wt or SnRV Env^mut^), or not. The cultures were passaged for 18 days postinfection, stained with crystal violet, and imaged by light microscopy. Bar; 100 µm.

To examine if the SnRV wt molecular clone is infectious, we tested its ability to spread in BF-2 cells since this line is susceptible to SnRV [2,19]. We performed two independent spreading assays that measured the increase in viral products over time in the supernatants of the infected cultures (Fig 2A and 2B). One assay quantified the viral RNA levels by qRT-PCR using SnRV-specific primers (Fig 2A and S2 Table), and the other measured the reverse transcriptase (RT) activity by the SG-PERT assay [24] (Fig 2B). We used the parental virus secreted from the E-11 cells as a positive control. Negative control consisted of SnRV Env^mut^ - a SnRV wt clone derivative that harbors a frameshift mutation in its *env* gene (Fig 1B). We collected the supernatants of E-11 cells and of BF-2 cells electroporated with SnRV wt or SnRV Env^mut^ clones. We then infected naïve BF-2 cultures with equal amounts of the different SnRVs, normalized by qRT-PCR (Fig 2A) or by RT activity (Fig 2B). We passaged the infected cultures for 38 days, during which we periodically sampled a fixed volume of the culture supernatants for qRT-PCR (Fig 2A), or pelleted the virions from the culture supernatants and assayed their levels by SG-PERT (Fig 2B). The readouts of both assays showed that SnRV wt spread in the infected cells with kinetics similar to the E-11-derived virus, in contrast to the SnRV Env^mut^ that did not spread (Fig 2A and 2B; and see raw values for these figures and figures below in S3 Table). These results demonstrate the ability of the SnRV wt clone to infect and spread in BF-2 cells, similar to the parental virus originating from the E-11 cells.

Previous studies showed that infection of BF-2 cells by SnRV results in cytopathic effects (CPE), followed by a “mesh-like” appearance and induction of an elongated cellular shape in the chronically infected, surviving cells [2,19]. Indeed, upon culturing, the SnRV wt-infected BF-2 cells acquired the typical elongated shape. In contrast, SnRV Env^mut^-infected cells formed a flat and denser monolayer, resembling the appearance of the naïve BF-2 cell culture (Fig 2C). These results further support the notion that SnRV wt clone is infectious and induces a typical CPE in BF-2 cells.

### Substitutions of the 3’ORF with foreign sequences reduce SnRV RNA levels, virions, and infectivity

It has been hypothesized that the 3’ORF of SnRV encodes a Tat-like protein (GenBank AAC54864.1) [3] based on the presence of an N-terminal acidic region, cysteine cluster (CDHYCRCLNPPRFCWC; amino acids 103–118), and a small basic region (RKHKR; amino acids 119–123) - features of lentiviruses Tat proteins [9]. To test this hypothesis, we investigated the infection and transcription of SnRV 3’ORF mutants. We replaced the majority of the 3’ORF with either the EGFP (hereafter, ‘GFP’) coding sequences or the puromycin-N-acetyltransferase (*pac*) gene, generating the ‘3’ORF14GFP’ or the ‘3’ORF14Puro’ clones, respectively (Fig 1C). Notably, the lengths of the GFP and the pac genes (about 600 and 700 bp, respectively) are similar to the 3’ORF size (about 600 bp). In these clones, the first 14 codons of 3’ORF (including its initiator methionine) are fused to the GFP or pac genes (lacking their original initiator methionine codon), and the other 191 codons of the 3’ORF are deleted. We retained the first 14 codons of 3’ORF because they overlap with the envelope C-terminus end.

**Fig 3:**
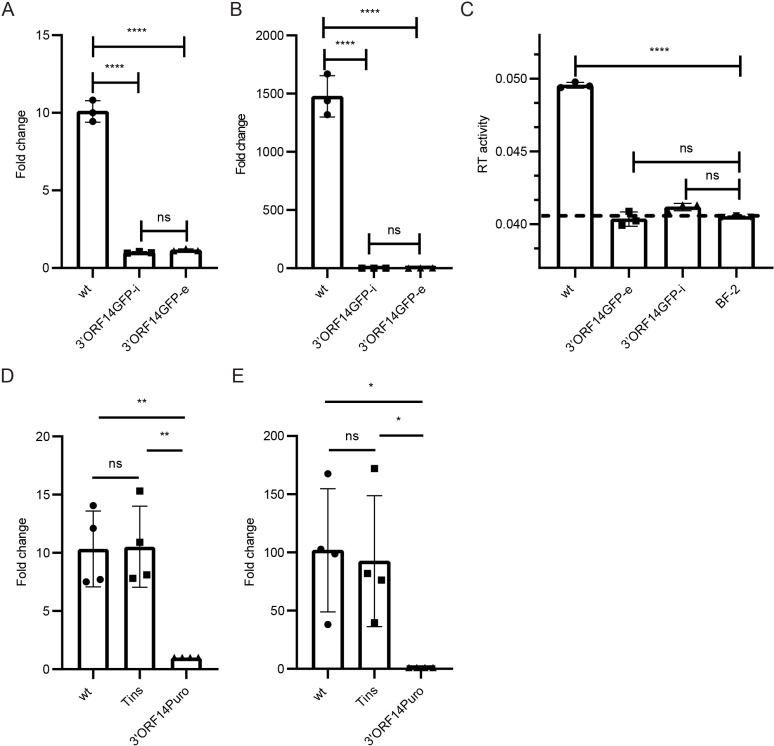
Effects of 3’ORF replacements on the levels of SnRV genomic RNA and virions. Genomic RNA levels (quantification by qRT-PCR with *pol*-derived primers) in cell extracts (normalized to actin RNA levels, A) or culture supernatant (140µl, B) of BF-2 cultures chronically infected with the wt virus (wt) or the 3’ORF14GFP clone (3’ORF14GFP-i), or stably transfected with the 3’ORF14GFP clone (3’ORF14GFP-e). (A, B) The graphs show the fold change in the SnRV genomic RNA levels in wt and 3’ORF14GFP-e relative to the 3’ORF14GFP-i (set to 1) cultures. Each bar represents the average of three technical repeats. ****p ≤ 0.0001, ns - not significant; one-way ANOVA. (C) SG-PERT assay for SnRV RT activity. The supernatants of the indicated cultures (10 ml each) were subjected to ultracentrifugation, and the RT activity of the pellets was determined as in Fig 2B. The dashed line marks the background level obtained from supernatant pellets of naïve BF-2 cultures (BF-2). Each bar represents the average of three technical repeats. (D, E) SnRV clones (wt, Tins, or 3’ORF14Puro) were electroporated into BF-2 cells, and three days posttransfection, the SnRV genomic RNA levels were quantified in cell extracts (D) and culture supernatants (E), by qRT-PCR and normalized to the transfection efficiencies. Transfection efficiencies were quantified by measuring the leaky transcription (in the fish cells) of the yeast *LEU2* gene, located in the pGREG525 plasmid backbone shared by the different SnRV constructs. Graphs show the fold change in the normalized genomic RNA of wt and Tins clones, relative to 3’ORF14Puro clone (set to 1); n = 4, * p ≤ 0.05, ** p ≤ 0.01, ns - not significant; one-way ANOVA.

First, we tested 3’ORF14GFP expression and infectivity. Transient transfection (by electroporation) of BF-2 cells with the 3’ORF14GFP clone yielded readily detected GFP^+^ cells (S1A Fig), suggesting the generation of viral long transcripts spliced into 3′ORF mRNAs as described before [3]. Incubation of naïve BF-2 cells with the supernatant of the transiently transfected culture resulted in only a few transduced (GFP^+^) cells (S1B Fig), with no expansion of the GFP signal over subsequent passages of the infected culture, indicating a lack of virus spread. We sorted by fluorescence-activated cell sorting (FACS) the GFP^+^ cells and expanded them to generate 3’ORF14GFP-infected (‘3’ORF14GFP-i’) culture, stably expressing the SnRV mutated genome (S1C Fig). An independent culture of BF-2 cells stably expressing the 3’ORF14GFP genome after electroporation (‘3’ORF14GFP-e’) was similarly generated. We then quantified the SnRV genomic RNA levels by qRT-PCR using primers derived from the *pol* gene. The cell-associated levels of the SnRV genome were significantly lower (~10-fold) in 3’ORF14GFP-i or 3’ORF14GFP-e cultures compared to BF-2 cells chronically infected with the wt virus (Fig 3A). The genomic RNA levels in the 3’ORF14GFP-i or 3’ORF14GFP-e culture supernatants barely raised above the background levels (ΔCt < 1.6; calculated for reverse transcription reactions with no RT, made to control for contaminating genomic DNA), and where three orders of magnitude lower, compared to wt-infected culture (Fig 3B). The reduction in 3’ORF14GFP extracellular genomic RNA levels may result from reduced virion production or reduced packaging in otherwise normal levels of assembled virions. To answer this, we pelleted the virions from the culture supernatants and quantified their levels by SG-PERT (Fig 3C). In contrast to the SnRV wt that showed a significant RT activity over the background levels, 3’ORF14GFP-i and 3’ORF14GFP-e culture supernatants had almost no such activity. Thus, the reduction in 3’ORF14GFP extracellular genomic RNA levels correlates with poor virion generation and release. The negative impact of replacing the 3′ORF sequence on the intracellular and extracellular genomic RNA levels was similarly evident for the 3’ORF14Puro clone in transient expression experiments: the genomic RNA levels of this clone were significantly lower than the wt clone - both intracellularly (~10-fold average reduction; Fig 3D) and extracellularly (~100 fold; Fig 3E). Altogether, these results indicate that replacing the 3′ORF with heterologous sequences greatly impairs SnRV expression, release, and infectivity.

### The 3’ORF product has a minimal effect on SnRV replication, expression, and morphology change in BF-2 cells

The above defects may stem from substituting 3’ORF nucleotides with foreign sequences, the absence of the 3’ORF protein product, or both. To investigate the potential contribution of the 3’ORF protein product to virus replication, we made a frameshift mutation in the 3’ORF sequence by a single-nucleotide (T) insertion, generating an internal stop codon (downstream of the first 14 codons of 3’ORF). The resulting clone (named ‘Tins’; Fig 1C) had only a minimal change in the 3’ORF nucleotide sequence (compared to the above sequence replacement mutants) but expressed a short truncated version (14 residues long) of the 3’ORF product. Tins clone showed normal intracellular and extracellular genomic RNA levels (Fig 3D and 3E).

**Fig 4:**
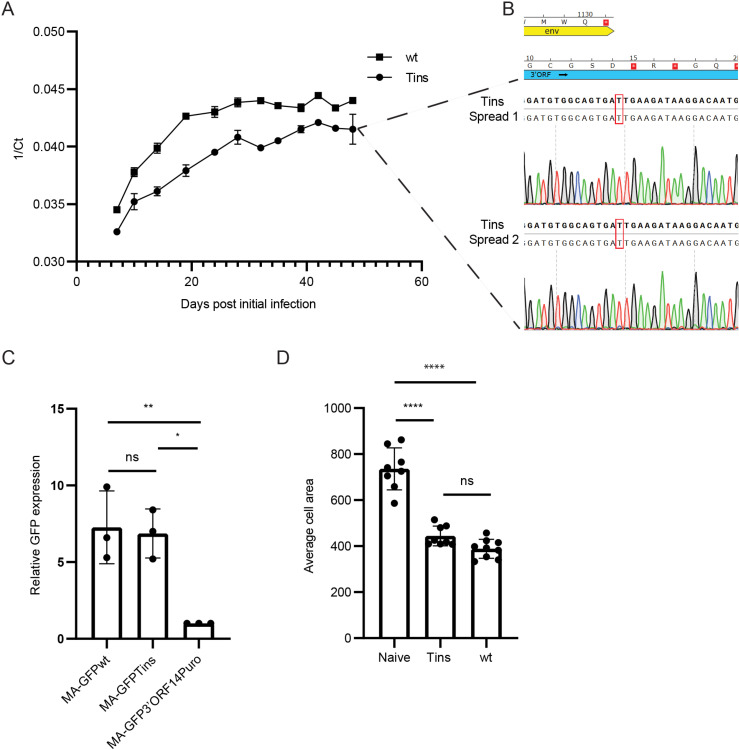
Spreading, expression, and induction of an elongated phenotype of wt and Tins clones in BF-2 cells. (A) Spreading of wt and Tins clones in BF-2 cultures. The levels of the viral genomic RNA in the culture supernatants were quantified by qRT-PCR and are presented as the average, with standard error bars, of the 1/Ct values of two samples per each time point from two independent spreading assays. (B) Sequence chromatograms from the two independent spreading assays (Spread 1 and 2) of the Tins clone. Supernatants of Tins-infected cultures from (A) were collected 48 days after the initial infection. The region containing the overlap between the env (yellow) and 3’ORF (blue) genes and the T insertion was amplified by RT-PCR and sequenced. Bold and regular letters represent the expected and the actual sequences, respectively. Red rectangles mark the inserted T. Red squares with white asterisks represent stop codons. Amino acids appear in a 1-letter code. (C) MA-GFP expression of SnRV MA-GFPwt, MA-GFPTins, or MA-GFP3’ORF14Puro clones. Equal amounts of the indicated clones were electroporated into BF-2 cells together with a mCherry-expressing plasmid (transfection efficiency control). Three days posttransfection, the transfected cultures were analyzed using flow cytometry for GFP and mCherry signals. The graph depicts the GFP expression levels (normalized to mCherry expression) of the indicated clones relative to the MA-GFP3’ORF14Puro clone (which was set to 1); n = 3. (D) Naïve, or wt or Tins -infected BF-2 cells were stained and imaged, and their average cell area was calculated. 300-500 cells were monitored for each kind of cell. * p ≤ 0.05, ** p ≤ 0.01, **** p ≤ 0.0001; one-way ANOVA.

Next, we compared the spreading of the wt and Tins clones in BF-2 cells. We transiently transfected the clones in BF-2 cultured cells, collected the virus-containing supernatants of the cultures three days post-electroporation, and infected naïve BF-2 cultures with equal amounts of viral RNA genomes, normalized by qRT-PCR. The infected cultures were passaged for up to 48 days while culture supernatants were sampled. SnRV genomic RNA was extracted from the samples at the indicated time points and quantified by qRT-PCR. Analysis of two experimental repeats for each clone revealed that the Tins clone spread in the BF-2 cells, albeit with slightly delayed kinetics compared to the wt clone (Fig 4A). RT-PCR and sequencing of the amplicons of the mutated region of the Tins clones at day 48 post the initial infection revealed the maintenance of the inserted T nucleotide and the downstream stop codons (Figs 4B and 1C).

Further analysis of the SnRV clones revealed that the viral genomic RNA levels in the cells and the culture supernatants, normalized to the cellular viral DNA levels, were nearly equal in the cells and slightly higher in culture supernatants for the wt virus compared to the Tins clone (about 2-fold; Spread I and II, Table 1). In transient transfection experiments, this difference in the culture supernatants was even more minor (1.1-fold) (Transient, Table 1). Altogether, these results suggest that although the 3’ORF product contributes slightly to the spreading of SnRV in BF-2 cells, this contribution is minor and, thus, is fundamentally different from Tat’s essential contribution to HIV replication [10].

**Table 1:** Genomic RNA levels of wt and Tins clones in spreading and transient expression assays in BF-2 cells.

		^a^ Viral RNA normalized to viral DNA	
Experiment	Clone	Cellular	supernatants
^b^ Spread I	wt	1.38 ± 0.04	2.4 ± 0.17
	Tins	1	1
^c^ Spread II	wt	1.04 ± 0.20	2.35 ± 0.05
	Tins	1	1
^d^ Transient	wt	0.99 ± 0.08	1.1 ± 0.13
	Tins	1	1

^a^ Quantified by qPCR (DNA genome) and qRT-PCR (RNA genome) with *pol*-derived primers. For each experiment, the viral RNA/DNA ratio of the wt clone was compared to that of the Tins clone, which was set to 1.

^b^ Quantified on day 48 of the spreading (shown in Fig 4A).

^c^ Quantified on day 47 of the spreading.

^d^ Quantified on day 3 after plasmid electroporation. n = 4.

In line with the above results, we found in a complementary experiment that the 3’ORF product was dispensable for efficient translation initiating at the matrix (MA) domain of the Gag precursor. To this end, we inserted a GFP coding sequence into the MA-CA junction in the *gag* gene (Fig 1D) of the wt, Tins, and the 3’ORF14Puro plasmids, generating ‘MA-GFPwt’, ‘MA-GFPTins’, ‘MA-GFP3’ORF14Puro’ clones (collectively named ‘MA-GFP’ clones). These clones express an MA-GFP fusion protein (terminating at a stop codon at the end of the GFP ORF); its expression depends on the translation initiation of the Gag precursor (at the MA domain) as the inserted GFP lacks the initiator methionine. Equal amounts of the MA-GFP clones were electroporated separately into BF-2 cells, together with a mCherry-expressing plasmid control. Three days post-transfection, the GFP signal (a proxy for Gag expression) and the mCherry signal (transfection control) were quantified by FACS. The MA-GFPwt and MA-GFPTins clones showed comparable GFP levels (normalized to mCherry levels) that were significantly higher (~7-fold difference) than that of the MA-GFP3’ORF14Puro clone (Fig 4C). The comparable GFP levels of the MA-GFPwt and MA-GFPTins clones further indicate that 3’ORF product does not have the same function as the HIV Tat protein, since the latter is essential for Gag protein expression [17].

We also noticed that in the Tins-infected BF-2 cultures, the cells acquired the typical elongated shape of SnRV-infected cells [2,19]. To quantify it, we infected or not naïve BF-2 cells with wt or Tins clones, passaged the cells (18 passages), fixed them, and stained the cultures with Alexa Fluor 568 phalloidin and DAPI to quantify the cells’ area (Materials and Methods). This analysis revealed a similar average cell area for the wt and the Tins-infected cells, which was significantly smaller than the average cell area of the naïve BF-2 cells (Fig 4D). Thus, viral factors other than the 3’ORF product should induce this phenotypic change.

### The SnRV promoter is versatile: robustly operates in fish and mammalian cells

**Fig 5:**
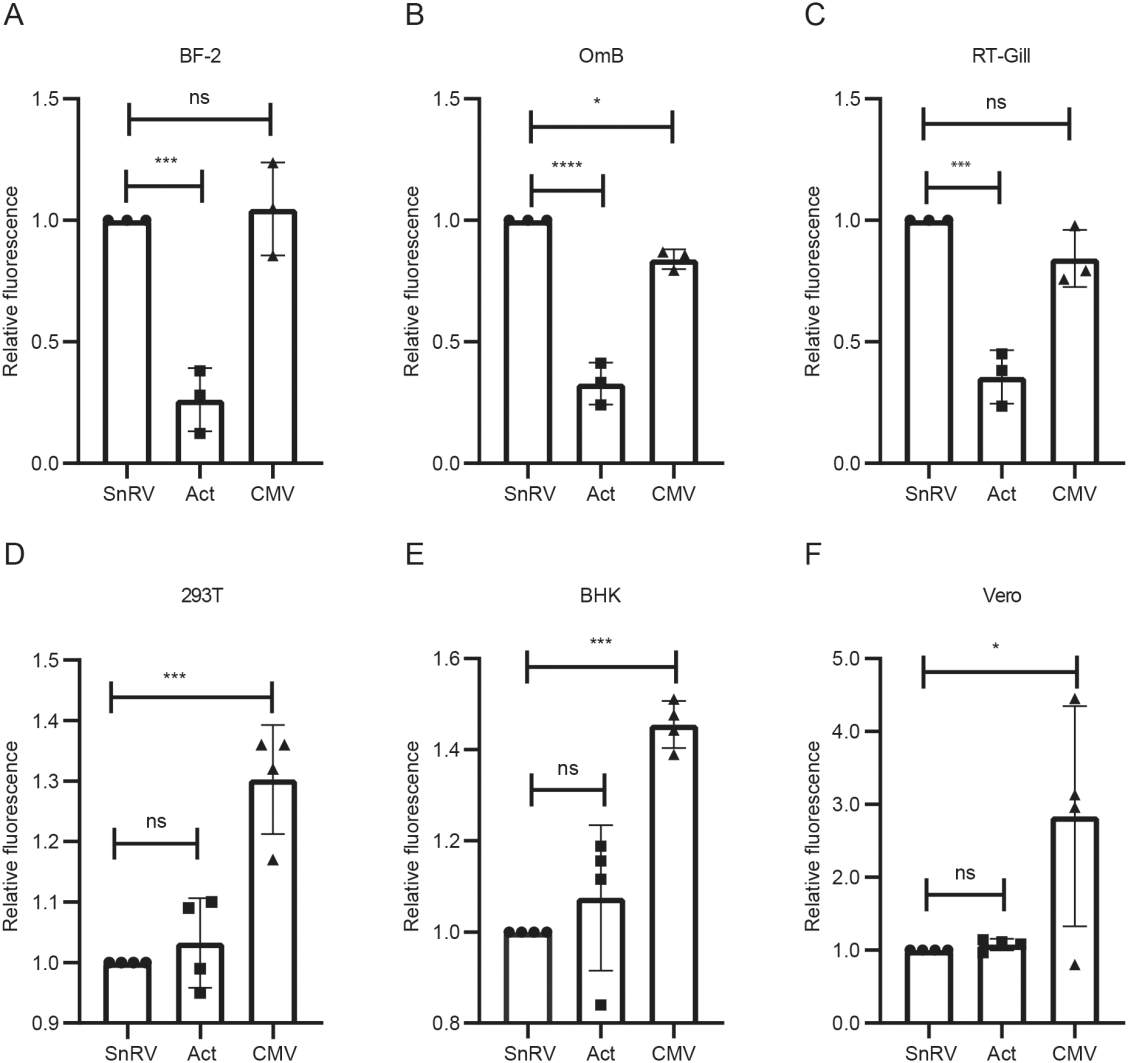
SnRV promoter activity in fish and mammalian cells. The indicated fish (A-C) or mammalian (D-F) cells were transfected with the GFP reporter gene under the control of the SnRV promoter (SnRV, circles), the tilapia β-actin promoter (Act, squares), or CMV promoter (CMV, rectangles). The Y-axes present the GFP mean fluorescence relative to the SnRV promoter sample (which was set to 1), quantified by FACS one day posttransfection, except for the RTgill-W1 cells (C) that were analyzed two days posttransfection. n = 3 or 4 for fish or mammalian cells, respectively. *p ≤ 0.05, ***p ≤ 0.001, ****p ≤ 0.0001, ns - not significant; one-way ANOVA.

The independence of the SnRV promoter activity from the 3’ORF product led us to test its expression independently of any other SnRV products. To this end, we cloned upstream of a GFP reporter gene the first 605 bp of the SnRV genome, containing the 5’ LTR (518 bp) and an adjacent short noncoding region (87 bp), generating the SnRV LTR-GFP plasmid. Next, we tested GFP expression in various fish and mammalian cells transfected with this plasmid, including the bluegill BF-2, tilapia OmB, rainbow trout RTgill-W1, human HEK 293T, hamster BHK-21, and monkey Vero cell lines. We quantified the SnRV promoter activity by measuring the GFP fluorescence using FACS. We compared it to two considerably strong mammalian and fish promoters - the cytomegalovirus (CMV) promoter and the tilapia β-actin promoter, cloned into the same plasmid backbone. In all the fish cells, the SnRV promoter showed comparable activity to the CMV promoter and significantly higher activity than the tilapia β-actin promoter (Figs 5A-C and S2A-C). In the mammalian cells, the fish promoters showed comparable activity, which was somewhat reduced (~1.3 for HEK 293T; ~ 1.5-fold for BHK-21; ~ 3-fold for Vero) compared to the strong CMV promoter (Figs 5D-F and S2D-F). Thus, the SnRV promoter can be versatile and robust in fish and mammalian cells.

### The SnRV promoter is active in zebrafish embryos as early as the blastula stage

**Fig 6:**
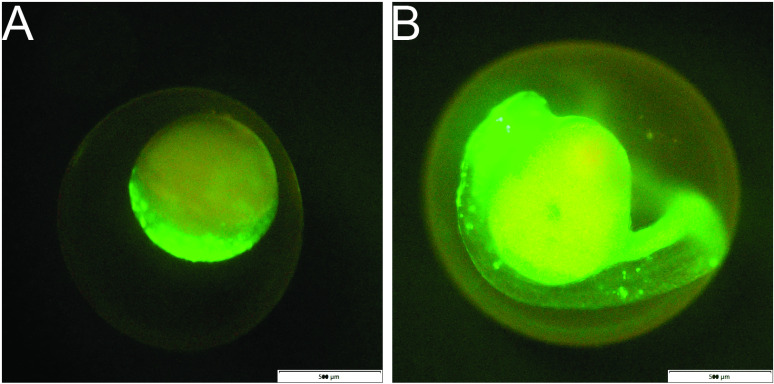
Transient expression of SnRV promoter in zebrafish embryos. The SnRV LTR-GFP plasmid was injected into zebrafish zygotes and monitored at one-hour intervals. Expression began at the blastula stage (A) and was continually evident throughout development and organogenesis (B). Bars = 500 µM.

The preceding results demonstrate that the SnRV promoter exhibits robust transcriptional activity across various cell lines independent of SnRV proteins. To assess the *in vivo* functionality of the SnRV promoter construct without any of the SnRV proteins, we initially microinjected the SnRV LTR-GFP plasmid into fertilized zebrafish (*Danio rerio*) eggs in the one-cell-stage embryos. We monitored the transient expression of GFP during embryonic development. In a fraction of injected embryos, we detected the GFP signal as early as the blastula stage (4–5 hours post-fertilization, hpf), immediately after activation of zygotic gene expression occurs (Fig 6A). At the pharyngula period (24–72 hpf), characterized by massive organogenesis, GFP expression was readily detected in the head and body (Fig 6B).

### Activity of the SnRV promoter in transgenic zebrafish

**Fig 7:**
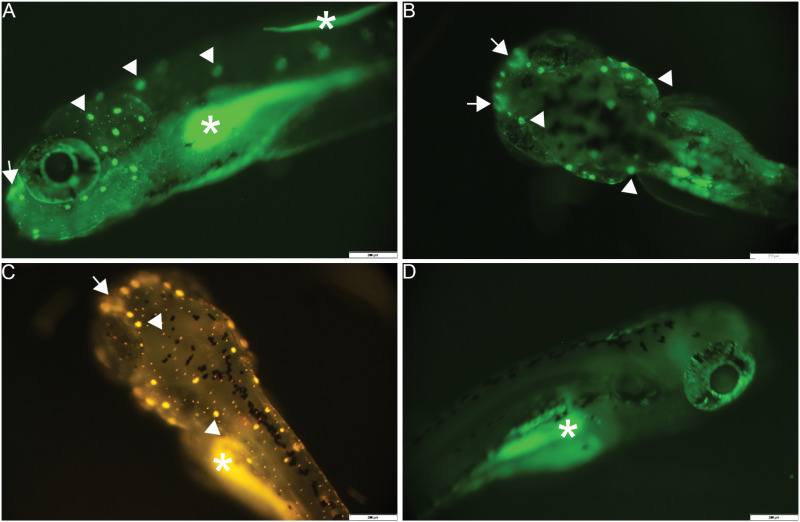
Expression of SnRV promoter in sensory organs of transgenic zebrafish larvae. Lateral (A, D) and dorsal (B, C) views of a 6 dpf *Tg(SnRVLTR:EGFP)* larva (A, B) or wt (not transgenic) siblings (C, D), showing GFP expression (A, B) and DASPEI signal (C) in the olfactory epithelium (arrows) and neuromasts hair cells (arrowheads). Non-transgenic larva was used for DASPEI staining due to the overlapping excitation and emission spectra of GFP and DASPEI. (D) Control, unstained larva imaged as in A and B. Asterisks mark regions with autofluorescence of the yolk sack. Bars = 200 µM.

The above results encouraged us to prepare transgenic lines with the SnRV LTR-GFP plasmid for further analysis of the spatiotemporal patterns of the SnRV promoter activity. Two independent *Tg(SnRVLTR:EGFP)* lines, tlv15 and tlv16, were generated, exhibiting identical expression patterns. Crosses of *Tg(SnRVLTR:EGFP)* males with wt females yielded 50% GFP-positive embryos, indicating the presence of a single genomic insertion of a functional transgene in the heterozygous males. A low level of GFP expression was first detected at 6–7 hpf and was readily detected at later stages - mainly in the olfactory epithelium and hair cells within neuromasts of the anterior and posterior lateral line system [25–27] (Fig 7A and 7B), the identity of which was verified using DASPEI staining of wt sibling (Fig 7C). No GFP or DASPEI signals were detected in these tissues in control, unstained wt sibling (Fig 7D).

**Fig 8:**
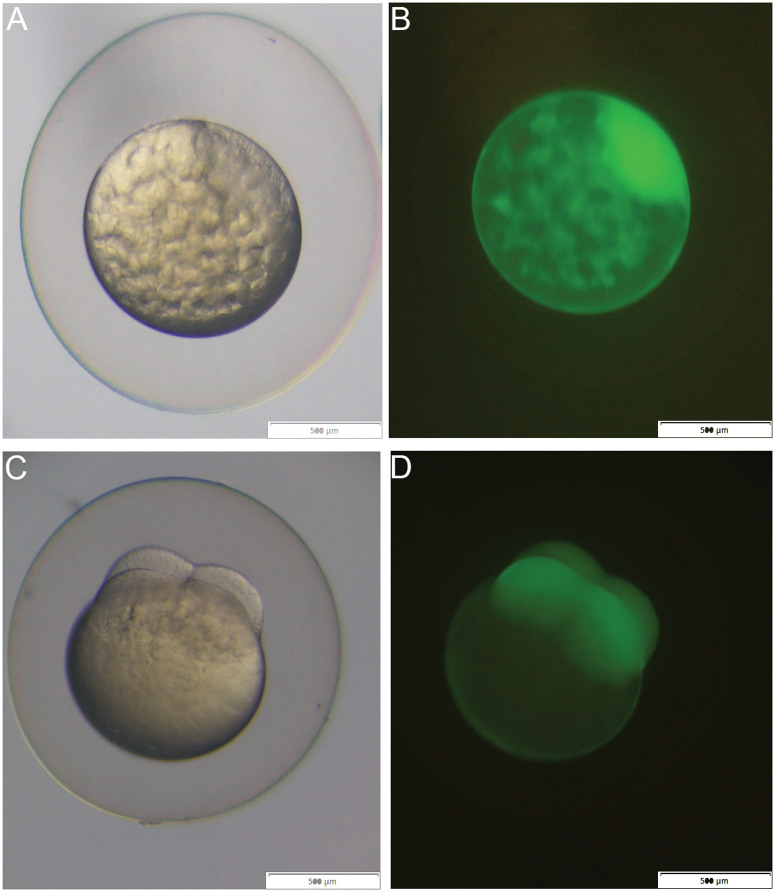
Maternal inheritance of the GFP reporter. Brightfield (A, C) and fluorescent images (B, D) of a fertilized zebrafish egg (A, B) and a four-cell stage embryo (C, D) derived from the transgenic female. Images depict the accumulation of GFP at the animal pole (B), and the blastomeres (D). Bars = 500 µM.

Crosses of *Tg(SnRV*LTR:EGFP) heterozygous females with wt males yielded 100% GFP-positive embryos, evident at the zygotic stage when cytoplasm streams towards the animal pole (Fig 8A and 8B), and the early cleavage stages (Fig 8C and 8D). The fact that all resulting embryos exhibited GFP positivity indicates maternal inheritance of the transgene product (GFP). Maternally inherited GFP slowly decayed, and by 14 days post fertilization (dpf), 50% of the embryos lost all of the GFP signal, further indicating maternal inheritance of the GFP. The other 50% of the offspring exhibited *de novo* expression of GFP in the anterior and posterior lateral line and olfactory epithelium, as described above in Fig 7. The GFP signal in the olfactory epithelium remained detectable in adults (see below).

**Fig 9:**
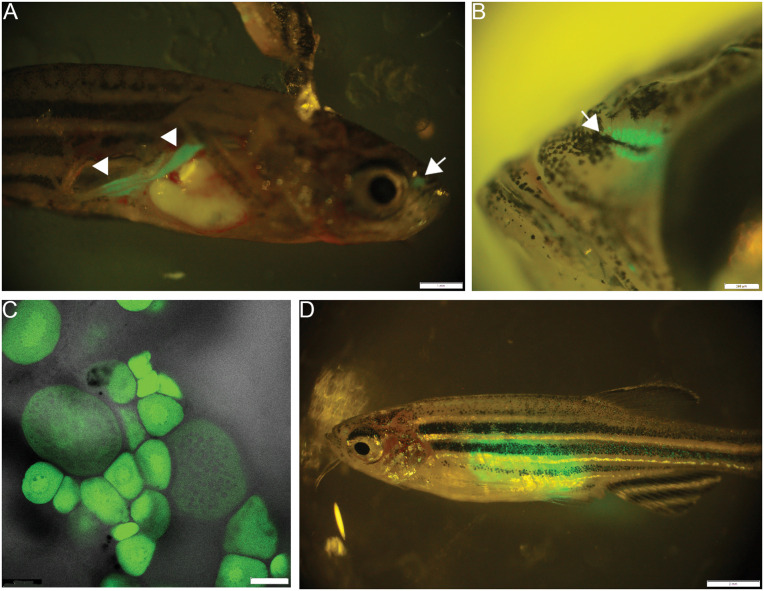
Expression of SnRV promoter in the adult olfactory epithelium and gonads. Lateral views of dissected (A) and undissected (B) transgenic males, showing GFP signal in the testis (A; arrowheads) and the olfactory epithelium (A, B; arrows). (C) An isolated ovarian biopsy showing a GFP signal in oocytes of different developmental stages. (D) A lateral view of an undissected adult transgenic female showing the ovarian-generated GFP signal.

At the adult stage, in addition to the expression in the olfactory epithelium (Fig 9A and 9B), strong GFP expression was apparent in the gonads (Fig 9A, 9C and 9D). Specifically, in the male, GFP expression was detected in the testis upon dissection and exposure of the internal organs (Fig 9A). In the female, and consistent with the maternal inheritance of SnRV promoter-derived reporter, expression of GFP was detected in the oocytes within the ovary (Fig 9C). The GFP signal was readily detected in early oocytes, indicating expression by the oocyte at this stage. Remarkably, the strong GFP signal in the ovaries could be detected by external examination of the adult females without the need for dissection (Fig 9D).

## Discussion

SnRV possesses features of complex retroviruses, including a relatively long cytoplasmic tail of the envelope protein, complex splicing patterns, and the presence of ORFs for several putative accessory/regulatory proteins. The product of one of these ORFs - the 3’ORF - has three domains (an N-terminal acidic region, a cysteine cluster, and a basic region) shared by lentiviruses’ Tat proteins and, accordingly, was speculated to serve as the SnRV Tat [3]. We constructed an infectious molecular clone to test this hypothesis, which showed comparable replication kinetics with its parental SnRV derived from E-11 cells. This result and the identity between the sequence of the molecular clone and the E-11-derived virus - revealed by the mass spectrometry analyses - imply that the SnRV wt clone authentically represents its parental virus. Thus, the differences between the sequence of the SnRV molecular clone and the GenBank sequence likely stemmed from sequencing mistakes of the latter.

In contrast to the SnRV wt clone, 3’ORF14Puro or the 3’ORF14GFP mutant clones, harboring substitutions of the 3’ORF with foreign sequences (pac or GFP, respectively) of similar lengths, showed greatly reduced viral expression and production. While this observation supports a Tat-like function for the 3’ORF product, an out-of-frame point mutation in 3’ORF (leaving only 14 out of the 205 predicted residues in the cognate Tins clone) had only minimal effect on SnRV replication. Moreover, the maintenance of the inserted T nucleotide (and the downstream stop codons) along 48 days of viral replication implies that no strong selection for reversion to the wt 3’ORF sequence occurred. Altogether, these results strongly suggest that the 3’ORF product is not a major transactivator of SnRV transcription. This notion also distinguishes SnRV from another phylogenetically related virus - the human foamy virus (HFV, a prototype of the *Spumaretrovirinae* subfamily). HFV possesses the *tas* gene with a genomic organization similar to the SnRV 3’ORF (the *tas* gene start has a short overlap with the *env* gene end). Still, HFV expression strongly depends on the *tas* gene product - the Tas (Bel-1) protein, which trans-activates transcription from HFV LTR and an internal promoter [28–30] in contrast to the lack of substantial effect of 3’ORF deletion on SnRV replication. In addition, we observed that SnRV wt and Tins clones similarly induced the unique elongated shape in BF-2-infected cells [2,19]. It is unknown if this phenotypic change reflects cellular transformation and what viral factors induce it, but our observation suggests that the 3’ORF product does not function in this phenotypic transition.

We quantified several defects for the 3’ORF14Puro and the 3’ORF14GFP mutant clones: substantially reduced cellular and extracellular genomic RNA levels in both transient (3’ORF14Puro) and stable (3’ORF14GFP) expression conditions, reduced Gag expression (inferred from the reduced expression of a reporter GFP gene, inserted in-frame with Gag’s MA domain), drop in virion production (no detectable RT activity in culture supernatants), and infectivity (negligible transduction of the 3’ORF14GFP clone). The reason for these replication defects of the 3’ORF14Puro or 3’ORF14GFP mutant clones is currently unknown but likely is the result of *cis*-acting effects: either alterations to the viral RNA fold introduced by the foreign sequences, the absence of an essential *cis*-acting element(s) overlapping the 3’ORF sequence, or both. Accordingly, the near-normal replication of the Tins clone may reflect a minimal impact of its single-point mutation on such putative *cis*-acting function of the 3’ORF sequence. In line with this notion, *cis*-acting elements close to the 3’ end of the viral genome and affecting cellular and extracellular viral RNA levels are documented for other retroviruses [31]. Examples include the constitutive transport element (CTE) of the Mason-Pfizer monkey virus (MPMV), which binds the NXF1(TAP)/NXT1 cellular factors to enhance nuclear export of unspliced viral RNAs [32,33], or the 115-nucleotide direct repeat (DR) that flanks the v-*src* gene in the Rous sarcoma virus. DR mutations have complex effects, reducing the levels of viral RNA nuclear export, stability, cytoplasmic accumulation, packaging, and particle assembly [34–37]. Future studies should elucidate the *cis* function, if any, of the 3’ORF sequence. Such experiments may involve the transfer of the 3’ORF sequence into heterologous reporter systems (e.g., [32]), other locations in the SnRV genome, or inverting it in its original location. In addition, our results do not highlight a clear function for 3’ORF activity needed in *trans*. Such a putative function is perhaps required for SnRV infection *in vivo* (e.g., regulating host gene expression or combating antiviral host factors). Comparing the replication of SnRV wt to 3’ORF mutants in fish may highlight such a requirement.

The transcriptional robustness of the SnRV promoter could be inferred from recent findings demonstrating that in E-11 cells, a single copy of the SnRV provirus transcribes ~5–6% of the cellular transcriptome [21]. Here, we showed that the expression from the SnRV promoter in the absence of any other SnRV proteins was robust in various fish cells and matched or exceeded the expression derived from strong mammalian and fish (CMV or β-actin) promoters. These results are consistent with the above notion that SnRV promoter does not require a viral transactivator, further demonstrating its dependency on host-cell transcription factors, as is the case for simple retroviruses [38]. The enhancers/promoters of these viruses are relatively short and packed with multiple (sometimes overlapping) transcriptional factor binding sites. Indeed, the SnRV LTR harbors putative binding sites for ~200 eukaryotic transcription factors (inferred by the PROMO server/TRANSFAC [39,40]). The specific transcription factors operating on the SnRV LTR in the different tissues, including the sensory organs and gonads, are yet to be determined. Examples include zebrafish ovarian transcription factors, for which sequence analyses of the SnRV LTR revealed multiple binding sites. These include Foxl2a and Foxl3 (expressed during early ovary differentiation), and Foxl2b (enriched in adult ovaries) [41], which bind the forkhead-box motif: 5′-RYMAAMA-3′ [42]. This motif repeats four times in the SnRV U3 sequence. The oocyte-specific transcription factors Figla and Nobox [43] that bind the sequences 5′-CANNTG-3′ (found five times in the SnRV U3 and R sequences) and 5′-TAATTA-3′ (found once in the SnRV U3 sequence), respectively. Figla and Nobox potentially interact with the oocyte-specific LIM-homeobox Lhx8a [44], which binds the 5′-TGATTG-3′ sequences [45]; one copy of this motif is present in the SnRV U3 sequence. These factors may contribute to the robust expression of the SnRV promoter in the ovary. In addition, at least part of the transcription factors operating on the SnRV promoter are likely conserved among fish and mammalian cells since the SnRV promoter activity was comparable to that of the CMV promoter in 293T or BHK cells. Of note, while the U3 region in the LTR is the primary promoter for retroviruses, at this stage, we cannot exclude the possibility that additional sequences in the SnRV LTR and the adjacent 87 bp-sequence also contribute to the SnRV promoter activity.

The induction of LTR transcription by hormones is a known phenomenon. For example, a hormone response element required for glucocorticoid induction was identified in the LTR of the mouse mammary tumor virus (MMTV) [46–49]. Transcriptional activation of LTRs in mouse endocrine tissues, including gonads (ovaries and testis), was also reported for a subset of ERVs - the VL30 retrotransposons [50]. This latter work demonstrated cAMP-mediated transcriptional activation of VL30 LTRs by gonadotropins, and the activity of hormone-activated enhancers within the LTRs. SnRV activity in the zebrafish gonads may also be hormone-dependent. However, while VL30 RNA is not detected in the oocytes (but is restricted to the external theca cell layer of the preovulatory follicle), the fact that we detected a robust GFP signal within the early zebrafish oocytes suggests a mode of transcriptional regulation different from that of the VL30 LTRs. Phylogenetic analyses revealed ERVs in reptiles, birds, amphibians, and fish lineages (including zebrafish) that are related to SnRV and that, together, consist of the SnRV-like clade [6–8]. It would be interesting to investigate if such ERVs show expression patterns similar to SnRV and if similar regulatory mechanisms dictate their tissue-specific expression.

The pattern of SnRV promoter expression revealed in zebrafish may highlight SnRV modes of transmission, assuming that similar patterns occur in naturally infected fish. Specifically, the robust expression of the promoter in tissues exposed to the surroundings, namely the olfactory epithelium and the lateral line system, may assist SnRV horizontal spreading through water. Moreover, it has been proposed that in mice, frequent germline acquisitions of new proviruses are mediated by the expression of the viruses in cells surrounding the oocytes[51–53]. Perhaps the SnRV expression in the fish gonads contributed to vertical transmissions and the establishment of the SnRV-like clade.

The GFP expression from the SnRV promoter was robust in the oocytes of adult transgenic females. The fact that all embryos, generated from crosses of heterozygous transgenic females with wt males, were GFP-positive, together with the fact that in half of these embryos the GFP signal slowly decayed, indicates that the GFP was maternally inherited. Thus, the GFP expressed in the oocytes lasted the period between fertilization, where the cells in the blastula are not expressing their mRNA [54], and the *de novo* transcription activation of the SnRV promoter (observed at 7 hpf in the progeny of the transgenic males). Accordingly, the SnRV promoter may be utilized to study the role of specific maternally inherited factors, by their over-expression or antisense-mediated downregulation.

The use of retroviral vectors can further enhance the above procedures. Commonly employed retroviral and lentiviral vectors predominantly utilize LTRs derived from simple murine leukemia viruses (MLVs) and the complex HIV, respectively. MLV and HIV LTRs facilitated the expression of vectors and their transgenes across various species and cell types. Subsequent enhancements led to the development of self-inactivating (SIN) vectors, wherein internal deletions rendered the LTRs inactive, necessitating transcription of the gene of interest to rely on internal promoters (for a review on retroviral vector evolution, see [55]). MLV- and HIV-based vectors have also been utilized to transduce fish cells and generate transgenic fish - see examples in [56–58]. Similarly, SnRV could serve as a platform for efficient transduction of fish cells. The robust activity of the SnRV promoter in HEK 293T cells, a primary cell line for producing MLV and HIV-based vectors, is anticipated to facilitate the production of SnRV-based vectors. However, achieving high titers of SnRV particles from helper plasmids is essential for the efficiency of such a system. Alternatively, the SnRV promoter, or its U3 region, could function as an internal promoter within MLV or HIV vectors to transduce fish cells *in vitro* or *in vivo*, thereby enabling efficient expression of foreign genes with SnRV tissue specificity.

In summary, we revealed that the SnRV promoter operates robustly and independently of other SnRV proteins. Its vigorous activity in mammalian and fish cells, *in vivo* expression patterns, and the fact that its expression products are maternally inherited should promote its use in designing versatile expression vectors for research and biotechnology usages.

## Materials and methods

### Cell cultures

E-11 cells (generously provided by M. Ucko, Israel Oceanographic and Limnological Research), BF-2 cells (ATCC no. CCL-91), OmB cells (generously provided by Dietmar Kültz, University of California Davis, USA) [59], and RTgill-W1 were grown at 25°C (E-11, BF-2, and OmB) or 20°C (RTgill-W1) in Leibovitz L-15 Medium (Biological Industries, 01–115-1A), containing 10% fetal bovine serum (FBS, Gibco, 10270–106), HEPES (10mM), L-glutamine (3.2 mM), and penicillin-streptomycin-nystatin solution (Biological Industries, 03–032-1C, diluted 1:250). HEK 293T, BHK-21, and Vero cells were grown in Dulbecco’s modified Eagle’s medium (GIBCO, 41965–039) supplemented with 10% fetal calf serum, l-glutamine, penicillin, streptomycin, and nystatin, at 37°C and 5% CO2.

### Construction of SnRV clones by homologous recombination in yeast

Genomic DNA of E-11 cells was extracted with GenElute Mammalian Genomic DNA Miniprep Kit (Sigma-Aldrich, G1N70), and four overlapping PCR fragments spanning the SnRV provirus, were amplified by PCR, using Phusion Hot Start II DNA Polymerase (Thermo Fisher Scientific, F549S). This PCR also added to the provirus termini sequences homologous to the sequences located upstream and downstream of the PvuII restriction site in the pGREG525 yeast plasmid [22]. The overlapping PCR fragments were co-transformed into yeast cells (Strain BY4741) with a PvuII-digested pGREG525. Plasmids with the SnRV genome, assembled by homologous recombination, were isolated from colonies that grew on SD plates lacking leucine. The plasmids were propagated in *E.coli*, and their sequence was verified. Additional mutant SnRV clones were similarly generated using homologous recombination in yeast by replacing the indicated wt sequence with a cognate PCR-generated mutant sequence.

### Construction of promoter-reporter constructs

The pT2-aanat2:EGFP-2A-Δclocka-5 × MYC construct has the backbone of the Tol2 transposable element-containing vector [60]. An XhoI-NdeI fragment (4.3 Kb) of this plasmid was replaced (by Gibson assembly reaction) with an XhoI-NdeI PCR fragment (1.4 Kb; amplified from a previous SnRV-LTR-EGFP construct), harboring the SnRV promoter and the EGFP gene to generate the SnRV LTR-GFP plasmid. In this plasmid, the EGFP gene (720 bp) and an upstream multiple cloning site (35 bp), are located downstream of the 5’ end (605 bp) of the SnRV genome. These 605 bp consist of the SnRV LTR (518 bp) and the adjacent downstream viral noncoding region (87 bp). SnRV LTR-GFP plasmid was digested with XhoI and SalI restriction enzymes to remove the SnRV sequence and to clone into these restriction sites the CMV enhancer/promoter (584 bp) or the tilapia β-actin promoter (1641 bp) [61].

### Sequencing

High-throughput sequencing (HTS) of SnRV RNA genome was accomplished as follows: RNA was extracted from SnRV virions, purified from supernatants of E-11 cells by ultracentrifugation through sucrose cushions, as described before for the tilapia lake virus (TiLV) [20]. RNA sequencing was performed at the W.M. Keck Center for Comparative and Functional Genomics (University of Illinois at Urbana-Champaign) using Roche 454 sequencing. The resulting contigs were assembled using the Newbler software (version 2.6). Sequencing of SnRV provirus and other clones was performed at the DNA Sequencing Unit at the G.S. Wise Faculty of Life Sciences, Tel Aviv University, using BigDye Terminator Cycle Sequencing Kit and 3500xL Genetic analyzer (Applied Biosystems).

### Mass spectrometry

Mass spectrometry analyses were performed at the Smoler Proteomics Center, Technion, Israel, as described before [23] and at the De Botton Institute for Protein Profiling, Weizmann Institute of Science, Israel. SnRV peptides were identified in the mass spectrometry data generated for TiLV virions as described before [23]. The peptides were aligned to the sequence of the SnRV wt clone using the SnapGene software.

### Transfection and infection of cells and SnRV detection

Mammalian (HEK 293T, BHK-21, or Vero) and fish (BF-2 or OmB) cells were transfected with the indicated plasmids using PolyJet (SignaGen Laboratories, SL100688) or Lipofectamine 2000 (Invitrogen, 52758), respectively. For electroporation, 5x10^5^ BF-2 or RTgill-W1 cells were electroporated using Neon transfection system (Invitrogen, MPK10096) and 5 µg of the indicated plasmid DNA with the following conditions: 100 µl kit, 1600 volts, 20 miliseconds, single pulse. When indicated, 1 µg of a plasmid expressing the mCherry marker (under the control of the CAG promoter) was co-electroporated to quantify transfection efficiencies. Immediately after the electroporation, the cells were seeded in 2 ml complete growth medium without antibiotics in one well of a six-well plate. The next day the culture media was replaced with fresh complete medium with antibiotics. Three days posttransfection, the supernatant was collected, filtered (0.45 µ) and used to infect naïve BF-2 cells (~ 50% confluency in 1 well of a six-well plate), in the presence of 4 µg/ml polybrene. The next day, the medium was replaced with a fresh complete growth medium. To detect SnRV RNA in supernatants of transfected or infected cell cultures, RNA was extracted from 140 µl of filtered (0.45 µ) supernatant with QIAamp Viral RNA Mini Kit (QIAGEN, 52904), treated with Baseline-ZERO DNase (Epicenter, DB0715K). Cellular SnRV RNA was extracted with EZ-RNA kit (Biological Industries, 20-400-100) or TRI Reagent (Sigma-Aldrich, T9424) and was treated with DNase. Extraction with the TRI reagent was also used to isolate genomic DNA of BF-2 cells, to normalized the SnRV RNA molecules to the SnRV provirus copies. First-strand cDNA synthesis was performed with qScript Flex cDNA Kit (Quantabio, 95049) and random primers. To monitor SnRV spread by qRT-PCR, *pol*-derived primers were used (S2 Table). To monitor SnRV spread by SG-PERT, about 10^6^ SnRV-infected BF-2 cells in 25 cm^2^ flasks were passaged for 38 days. Every 5 days, the infected cultures were spliited, and 10^6^ cells were re-seeded. At the indicated time points, virions were pelleted from 10 ml of culture supernatants by ultracentrifugation (150,000 x *g*, 1 h, 4 °C; with Optima XPN-80 ultracentrifuge, Beckman Coulter). Pellets of supernatants of naïve BF-2 cultures, obtained by ultracentrifugation, were used as background controls. Each pellet was resuspended in 100 µl of Leibovitz L-15 Medium with 10% FBS. 10 µl of the virion suspension were analyzed by SG-PERT [24] with modifications made to adapt the reaction to the dependency of SnRV RT activity on manganese [2]. Specifically, the 10 µl of the virion suspension were mixed with 50 µl of 1.2x RT reaction buffer [Tris-HCl, pH 8.3 (60 mM), MnCI_2_ (0.7 mM), NaCl (75 mM), IGEPAL CA-630 (0.06%), dithiothreitol (24 mM), dNTPs mix (240 nM), RNasin (Promega, N251B, 0.48 units), MS2 RNA (Roche, 10165948001, 0.29 µg), MS2 Fwd and MS2 Rev primers (S2 Table, 600 nM each]. The samples were incubated at 24 °C [2] for 1 h. 2 µl of the reaction were analyzed by qPCR, using the StepOnePlus Real-Time PCR System (ABI), qPCR reaction containing the MS2 Fwd and MS2 Rev primers (0.5 µM, each), and the Fast SYBR Green Master Mix (Thermo Fisher Scientific, 4385610). Notably, SnRV virion concentration greatly improved the signal-to-noise ratio of the SG-PERT readouts compared to unconcentrated particles. The above qPCR system was also used to quantify SnRV and cellular RNA in the indicated samples (0.5 µl), with primers listed in S2 Table.

### Detection of morphological changes in SnRV-infected BF-2 cells

To qualitatively detect morphological changes in SnRV wt or SnRV Env^mut^ -infected BF-2 cultures, the cultures were passaged for 18 days and stained with crystal violet. Cultures were then imaged by light microscopy (Axio inverted microscope and Axiocam ERc 5s camera; ZEISS). To quantify the morphology of naïve, wt-infected, or Tins-infected BF-2 cells, the cultures were passaged 18 times postinfection, fixed, and stained with Alexa Fluor 568 phalloidin (Invitrogen, A12380) and DAPI. For each of the three cell types, 8–9 fields (randomly chosen) were imaged with a spinning disk confocal (Yokogawa CSU-22 Confocal Head) microscope (Axiovert 200 M, Carl Zeiss MicroImaging), 10 × lens (NA 1.45, Zeiss) and Evolve or HQ2 (Photometrics) cameras. The total cell surface (deduced from the phalloidin signal) was divided by the total nuclei number (DAPI staining), and the average cell area was calculated using the SlideBook software.

### Flow cytometry

Electroporated cells were analyzed for GFP and mCherry fluorescence using the S1000EXi flow cytometry platform (Stratedigm), or the CytoFLEX S Flow Cytometer (Beckman Coulter). Total intensity of the GFP and mCherry signals were calculated multiplying the percentage of the fluorescently-positive cells by the mean intensity of the fluorescent signal and by subtracting the background fluorescence levels calculated for naïve cells.

### Generation of transgenic fish

Two transgenic lines, *Tg(snrvLTR:EGFP)*, registered in the Zebrafish Model Organism Database (ZFIN) as tlv15 and tlv16, were generated using the Tol2 system as described [62]. Tol2 transposase mRNA was synthesized *in vitro* using the mMESSAGE mMACHINE SP6 Transcription Kit (Ambion) and a linearized pCS-TP plasmid as a template. Approximately 1 nl of a DNA/RNA solution containing 25 ng/μl of the SnRV LTR-GFP plasmid and 25 ng/μl of Tol2 mRNA were injected into fertilized eggs at the single cell stage. Founder (F0) fish were raised to adulthood and outcrossed to screen for integration of the transgene into the germline. Transgenic GFP-expressing progeny (F1) of two independent founder fish were identified based on GFP expression and were raised to adulthood. Then, the two independent F1 fish were further outcrossed with wt fish to generate the F2 generation; 50% of the F2 offspring were GFP-positive, indicating one functional insertion.

### Neuromast staining

Staining of neuromast hair cells was performed as previously described [63] by exposing live 6 dpf zebrafish larvae to 0.005% 2-[4-(dimethylamino)styryl]-N-ethylpyridinium iodide (DASPEI; Molecular Probes, Eugene, OR) for 15 minutes. This short staining time was sufficient for the detection of the mitochondria-rich hair cells within neuromasts of the lateral line system with no background staining. Larvae were then anesthetized in tricaine and analyzed under an epifluorescence dissecting microscope (Olympus SZX12).

## Supporting information

S1 Fig Fluorescence and bright field microscopy of 3’ORF14GFP-transfected and infected BF-2 cultures. (A) 3’ORF14GFP-electroporated BF-2 cells, five days post-transfection. (B) A sub-confluent BF-2 culture was infected with the supernatant of the culture in (A), grown to confluency, and imaged six days postinfection. (C) 3’ORF14GFP-infected BF-2 cells from (B) were sorted by FACS, and the GFP^+^ cells were expanded to generate the 3’ORF14GFP-i culture. Bars = 400µM (A and B) and 200µM (C).
(TIF)

S2 Fig SnRV promoter activity in fish and mammalian cells - flow cytometry graphs. Dot plot graphs of a representative experiment described in Fig 5. The indicated fish (A-C) or mammalian (D-F) cells were transfected with the GFP reporter gene under the control of the SnRV promoter (SnRV), the tilapia β-actin promoter (Act), or CMV promoter (CMV). The X and Y axes present GFP fluorescence (GFP) and forward scatter (FSC), respectively, quantified by FACS one day posttransfection, except for the RTgill-W1 cells (C) that were analyzed two days posttransfection. GFP^+^ numbers represent the percentage of the gated GFP-positive cells.
(TIF)

S1 Table List of variations between the cloned/consensus SnRV sequence and the published SnRV sequence (GenBank U26458.1). (DOCX)

S2 Table List of primers and their applications. (DOCX)

S3 Table Values Behind Figures. (XLSX)
