# Supplementary figures and images for: The snakehead retrovirus promoter functions independently of the 3’ORF protein and its products are maternally inherited in transgenic zebrafish
Source: PLoS Pathog. 2025 Jun 12;21(6):e1013243. doi: 10.1371/journal.ppat.1013243 (PMC12193657; doi:10.1371/journal.ppat.1013243)

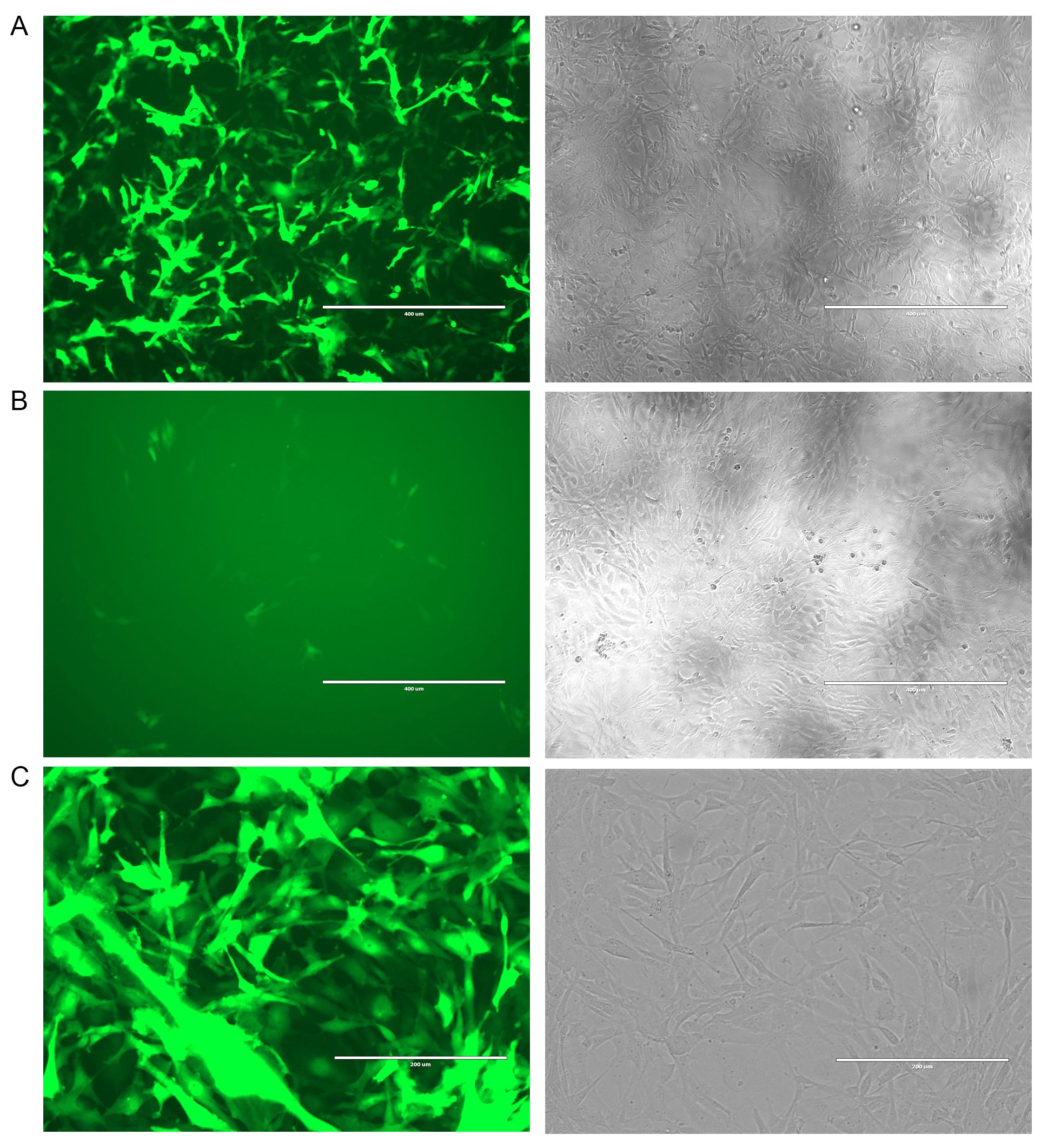

Supplement: S1 Fig — (A) 3’ORF14GFP-electroporated BF-2 cells, five days post-transfection. (B) A sub-confluent BF-2 culture was infected with the supernatant of the culture in (A), grown to confluency, and imaged six days postinfection. (C) 3’ORF14GFP-infected BF-2 cells from (B) were sorted by FACS, and the GFP+ cells were expanded to generate the 3’ORF14GFP-i culture. Bars = 400µM (A and B) and 200µM (C). (TIF) [file ppat.1013243.s001.tif]

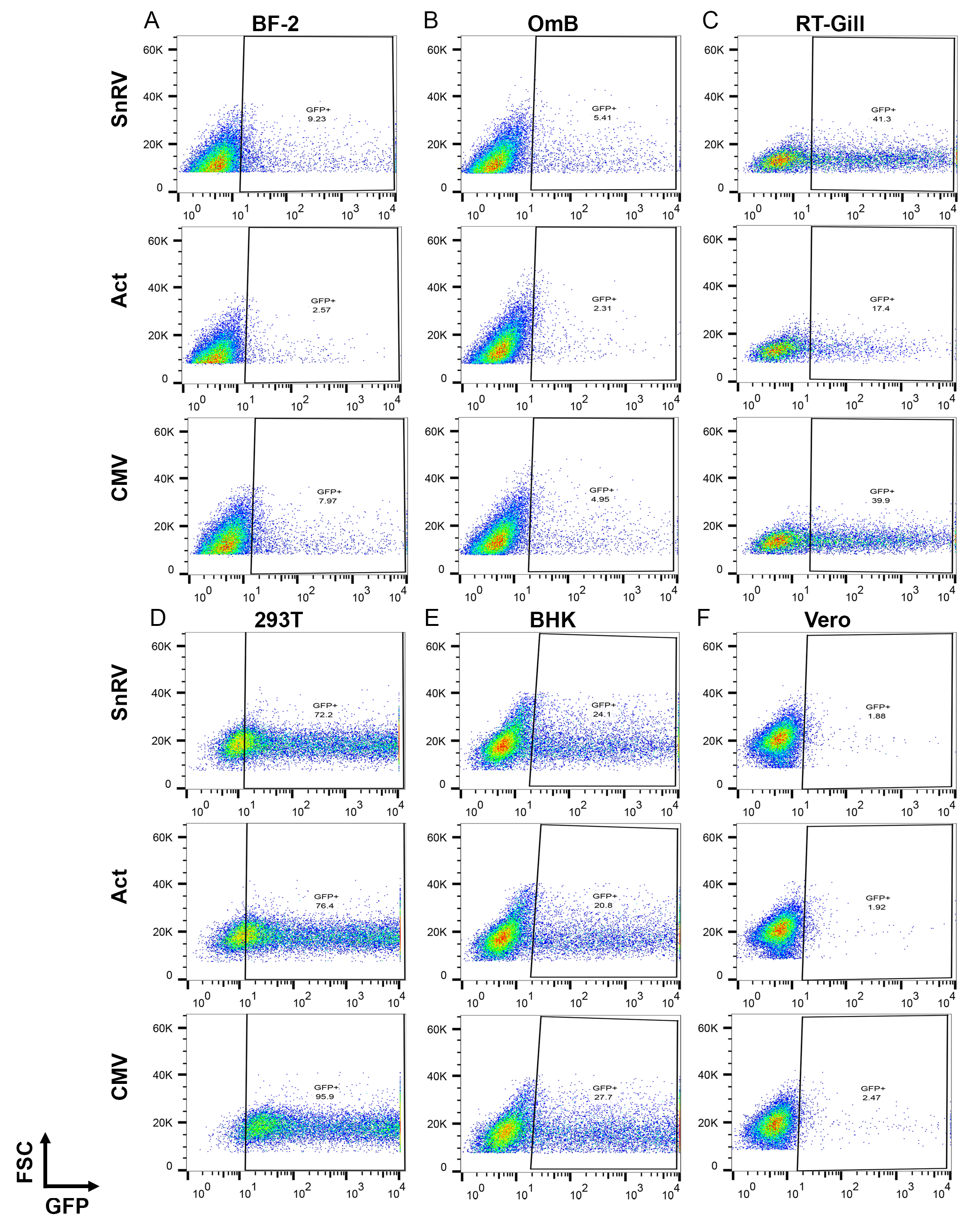

Supplement: S2 Fig — Dot plot graphs of a representative experiment described in Fig 5. The indicated fish (A-C) or mammalian (D-F) cells were transfected with the GFP reporter gene under the control of the SnRV promoter (SnRV), the tilapia β-actin promoter (Act), or CMV promoter (CMV). The X and Y axes present GFP fluorescence (GFP) and forward scatter (FSC), respectively, quantified by FACS one day posttransfection, except for the RTgill-W1 cells (C) that were analyzed two days posttransfection. GFP+ numbers represent the percentage of the gated GFP-positive cells. (TIF) [file ppat.1013243.s002.tif]
