# Supplementary material for: The snakehead retrovirus promoter functions independently of the 3’ORF protein and its products are maternally inherited in transgenic zebrafish
Source: PLoS Pathog. 2025 Jun 12;21(6):e1013243. doi: 10.1371/journal.ppat.1013243 (PMC12193657; doi:10.1371/journal.ppat.1013243)
Supplement: S1 Table — (DOCX) [file ppat.1013243.s003.docx]

**S1 Table.** List of variations between the cloned/consensus SnRV sequence and the published SnRV sequence (GenBank U26458.1)

| ^a^ Changes in SnRV wt clone | Affected noncoding and coding regions | ^b^ Peptides identified by mass spectrometry that overlap nonsynonymous substitutions |
| --- | --- | --- |
| G146A | Noncoding; downstream of the primer binding site |  |
| Deletion of A301 | Noncoding; upstream of the Gag ORF |  |
| C325G | Noncoding; upstream of the Gag ORF |  |
| C1210G | Gag (His to Asp) | QAWDYEGDEDP**D**VK;  QAWDYEGDEDP**D**VKEAEEEAWR  QAWDYEGDEDP**D**VKEAEEEAWRER |
| C1850A | Gag (Ala to Asp) | DQF**D**DKTGVSK |
| T3404C | Pol (Ile to Thr) |  |
| C4535T | Pol (Pro to Leu) |  |
| G4622C | Pol (Cys to Ser) |  |
| C4631T | Pol (Pro to Leu) |  |
| A4648C | Pol (Ile to Leu) |  |
| C5357T | Pol and ORF1 (Ser to Leu) | **L**AEGPCQK |
| G5452C | Pol (Val to Leu) |  |
| G5454C | Pol (Val to Leu) |  |
| T5775C | Pol (Ser to Ser) |  |
| G6523C | Env (Met to Ile) |  |
| A7601G | Env (Lys to Glu) | AEID**E**IRK |
| G8673A | Env (Arg to Gln) |  |
| G8849A | Env (Gly to Ser) | GHVLPFV**S**NSQEAPNTEIWEGLIEEAIR |
| G9285A | Env (Cys to Tyr) | EPPT**Y**QEWAR  QAPAYREPPT**Y**QEWAR |
| G9483A | Env and ORF2 (Gly to Glu) |  |

^a^Numbering according to the published SnRV sequence [[3]](https://sciwheel.com/work/citation?ids=9992156&pre=&suf=&sa=0&dbf=0).

^b^Peptides identified by mass spectrometry of purified SnRV virions produced in E-11 cells [[23]](https://sciwheel.com/work/citation?ids=3952749&pre=&suf=&sa=0&dbf=0) that overlap nonsynonymous substitutions (bold and underlined), in respect to the published SnRV sequence (GenBank file U26458.1).
