# Supplementary material for: The snakehead retrovirus promoter functions independently of the 3’ORF protein and its products are maternally inherited in transgenic zebrafish
Source: PLoS Pathog. 2025 Jun 12;21(6):e1013243. doi: 10.1371/journal.ppat.1013243 (PMC12193657; doi:10.1371/journal.ppat.1013243)
Supplement: S2 Table — (DOCX) [file ppat.1013243.s004.docx]

**S2 Table.** List of primers and their applications

| Primer | ^a^ Sequence (5' to 3') | Application |
| --- | --- | --- |
| SnRVGag-Pol qPCR F | GGATGGATCATGGAGGCTAAC | SnRV pol PCR amplification |
| SnRVGag-Pol qPCR R | GTTGAGGCTACTGTGGGATATG |  |
| MS2 Fwd | tcctgctcaacttcctgtcgag | SG-PERT |
| MS2 Rev | cacaggtcaaacctcctaggaatg |  |
| qPCR fish b-actin F | atctggcatcacaccttctac | BF-2 actin qPCR |
| qPCR fish b-actin R | tcttctccctgttggctttg |  |
| pGREG Leu Fw | tgaccctatcgccactatct | Leu qPCR |
| pGREG Leu Rev | ggtactgttggaaccacctaaa |  |
| 1_3'ORF14 GFP Fw | ACTGGTGAACGACACGACGG | Tins mutation PCR amplification |
| 3'ORF down BmgBI Rev | CGGTTTAAGTGTACCCTGAGCAC |  |

^a^Uppercase letters mark SnRV sequences; lowercase letters mark non-viral sequences.
